# Thermo-Mechanical and Creep Behaviour of Polylactic Acid/Thermoplastic Polyurethane Blends

**DOI:** 10.3390/polym14235276

**Published:** 2022-12-02

**Authors:** Yi-Sheng Jhao, Hao Ouyang, Fuqian Yang, Sanboh Lee

**Affiliations:** 1 Department of Materials Science and Engineering, National Tsing Hua University, Hsinchu 300, Taiwan; 2 Materials Program, Department of Chemical and Materials Engineering, University of Kentucky, Lexington, KY 40506, USA

**Keywords:** polylactic acid, thermoplastic polyurethane, creep, non-linear Burgers model, activation energy

## Abstract

There is a great need to develop biodegradable thermoplastics for a variety of applications in a wide range of temperatures. In this work, we prepare polymer blends from polylactic acid (PLA) and thermoplastic polyurethane (TPU) via a melting blend method at 200 °C and study the creep deformation of the PLA/TPU blends in a temperature range of 10 to 40 °C with the focus on transient and steady-state creep. The stress exponent for the power law description of the steady state creep of PLA/TPU blends decreases linearly with the increase of the mass fraction of TPU from 1.73 for the PLA to 1.17 for the TPU. The activation energies of the rate processes for the steady-state creep and transient creep decrease linearly with the increase of the mass fraction of TPU from 97.7 ± 3.9 kJ/mol and 59.4 ± 2.9 kJ/mol for the PLA to 26.3 ± 1.3 kJ/mol and 25.4 ± 1.7 kJ/mol for the TPU, respectively. These linearly decreasing trends can be attributed to the weak interaction between the PLA and the TPU. The creep deformation of the PLA/TPU blends consists of the contributions of individual PLA and TPU.

## 1. Introduction

Polylactic acid (PLA), a thermoplastic aliphatic polyester, has been regarded as a prospective and promising biodegradable material with the potential of replacing petrochemical plastics [1]. The biodegradable characteristics of PLA have attracted great interest for a variety of potential applications. However, the applications of PLA are limited by its brittleness, thermal stability, and impact resistance.

To increase the applications of PLA in a variety of fields, PLA-based composites and PLA-polymer blends have been developed. For example, Matta et al. [2] used melt blending to produce a biodegradable polymer blend consisting of PLA and polycaprolactone (PCL), which possesses better impact strength and toughness than respective PLA and PCL. Ho et al. [3] grafted PLA onto maleic-anhydride functionalized thermoplastic polyolefin elastomer to form thermoplastic polyolefin elastomer-grafted polylactide, which improves the toughness of PLA. Tokoro et al. [4] used three different bamboo fibers as PLA reinforcement materials to form PLA/bamboo composites, which exhibited higher bending strength and impact strength than PLA at room temperature.

Currently, most studies have been focused on tensile and impact deformation of PLA-based composites and blends [5,6,7,8,9]. There are few studies on the creep deformation of PLA-based composites and blends. Yang et al. [10] examined the effect of short bamboo fibers on the short-time creep of PLA-based composites and observed the increase of creep resistance with the increase of the weight fraction of bamboo fibers up to 60%. Georgiopoulos et al. [11] assessed the short-time creep deformation of PLA/PBAT (poly(butylene adipate-terephthalate)) blend reinforced with wood fibers and used Findley’s and Burger’s models in the creep analysis. Morreale et al. [12] studied the tensile creep of PLA-based bio-composites and found a strong dependence of the creep deformation on temperature and fabrics. Ye et al. [13] used a four-element Burger’s model in analyzing the creep of PLA prepared by the fused-filament process and observed the effects of the printing angle and stress. Waseem et al. [14] used the response surface methodology in the tensile-creep analysis of the PLA produced by three-dimensional printing for additive manufacturing. Guedes et al. [15] characterized the creep deformation and stress relaxation of PLA/PCL fibers and used Burger’s model and the standard solid model in the analysis. Niaza et al. [16] studied the long-term creep of PLA/HA (hydroxyapatite) composites and found that PLA/HA scaffolds under mechanical loading up to 10 MPa did not change shape and lose mechanical strength.

Thermoplastic polyurethane (TPU) has good elastic properties, transparency, and wear resistance. TPU is also biocompatible and bio-stable and is a promising material for a variety of implantable medical devices [17]. Blending TPU with PLA can alter the brittle characteristic of PLA and increase the toughness of PLA [18] due to the good elastic properties of TPU, while it is unclear if blending TPU with PLA can hinder or promote the motion of polymer chains under constant loading. This work aims to study the creep deformation of PLA/TPU blends. The effect of the mass ratio of PLA to TPU on the activation energies of the creep deformation of the blends is assessed.

It should be noted that there are extensive studies on the creep deformation of a variety of polymers, including polyurethanes [19,20]. The analysis has been based on the use of dashpot-spring models in describing the creep curves. However, there are little studies on the calculations of the activation energies of the rate processes for the creep deformation of polymers.

## 2. Experimental Details

The PLA (4032D, T_g_ around 55–60 °C) and TPU (300-grade series, ester type) used in this work were from Natureworks LLC (Minnetonka, MN, USA) and Bayer Co., Ltd. (Leverkusen, Germany), respectively. The PLA was a semi-crystalline polymer with 98% L-isomer and 2% D-isomer.

**Figure 1:**
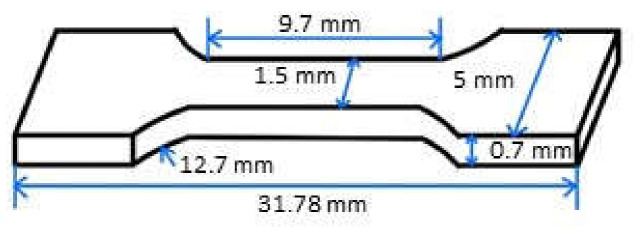
Schematic of the geometrical dimensions of specimens for creep tests.

PLA/TPU blends were prepared with different mass ratios of PLA to TPU (30/70, 50/50, and 70/30) via a melting blend method at 200 °C. Briefly, the PLA and TPU mixture of a preset mass ratio was dried in an oven at 80 °C for 4 h. The PLA/TPU mixture was heated to 200 °C to a molten state, which was then injected into a mold at 2.94 MPa to form a PLA/TPU plate. The plate was cooled down to 30 °C. Using laser cutting, specimens in a dumbbell shape, as shown in Figure 1, were prepared. The specimens were mechanically ground with CarbiMet papers of 400, 800, 1200, and 2500 grit, consecutively, and then polished with 1 μm alumina slurry. The polished specimens were annealed in the air in a furnace at 50 °C for 24 h to release the residual stresses introduced during the sample preparation and then naturally cooled down to room temperature in the furnace.

Thermal analysis of the prepared PLA/TPU blends was performed in a temperature range of −80 °C to 220 °C on a differential scan calorimeter (DSC) (Netzsch 200F3, Erich NETZSCH GmbH & Co., Selb, Germany). Nitrogen gas was used during the DSC scan to prevent the specimens from oxidation. The flow rate of nitrogen was 100 mL/min. Thermal cycling of the specimens of 8–10 mg in the mass loading was conducted with the heating and cooling rates of 10 °C/min and 40 °C/min, respectively, and 10 min each at 220 °C and −80 °C, respectively. The thermal cycling eliminated the thermal history of the specimens. After the thermal cycling, the specimens were reheated to 220 °C at a heating rate of 10 °C/min for thermal analysis. The glass transition temperatures, T_g_, of PLA, TPU, and PLA/TPU blends were determined to be the temperature at the midpoint of the corresponding heat-capacity jump in the heat-flow vs temperature plots.

The creep tests of the PLA, TPU, and PLA/TPU blends were performed on a dynamic mechanical analyzer (TA Q800 DMA, TA instrument, New Castle, DE, USA) in a temperature range of 10 to 40 °C. Due to the limitation of the instrument and the differences in the properties of the materials, the stresses applied to the PLA, TPU, and PLA/TPU blends were different. Before the creep test, each specimen was maintained at the preset temperature for 5 min to reach thermal equilibrium. The time for the creep tests was 40 min. After the creep test, the crept specimen was maintained in a stress-free state for 40 min. The strain was measured as a function of time during the test.

## 3. Results

**Figure 2:**
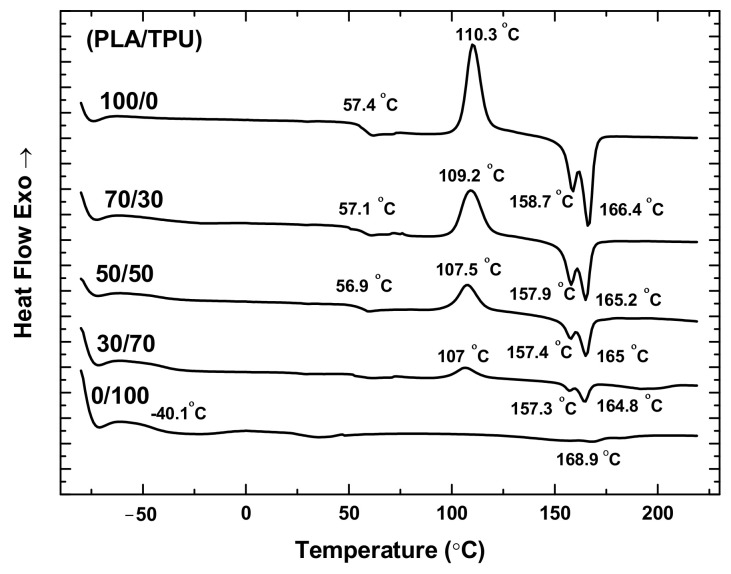
DSC curves of PLA, TPU, and PLA/TPU blends.

Figure 2 shows the DSC curves of the PLA, TPU, and PLA/TPU blends. For the PLA and TPU, the glass transition temperatures are 57.4 °C and −40.1 °C, respectively. For the PLA/TPU blends, there are two glass transition temperatures presented in the DSC curves with a weak one around −40 °C associated with the TPU glass transition temperature and the other one around 57 °C associated with the PLA glass transition temperature. Such a result suggests that PLA and TPU are thermodynamically immiscible, as supported by the dual melting for the PLA [17] and PLA/TPU blends [21,22]. It is interesting to note that the melting temperature of the PLA/TPU blends decreases with the increase of the mass fraction of TPU, revealing the contribution of TPU.

The dual melting behavior has been observed for many semi-crystalline polymers, including poly(butylene terephthalate), poly(ethylene terephthalate), and poly(ether ketone) [23,24,25,26]. The low-temperature endothermic peak represents partial melting of the “original” crystal, and the high-temperature endothermic peak represents the melting of “reorganized” crystals during the heating [27].

According to Figure 2, there is no exotherm peak of cold crystallization for the TPU in consistence with the amorphous structure of TPU. PLA is a semi-crystalline polymer with a slow crystallization rate. The rapid quenching of PLA from 220 °C before the DSC measurement does not allow the PLA to crystallize during cooling, i.e., the PLA polymer chains do not have enough time to migrate to equilibrium positions. During the heating, the polymer absorbs energy enabling the migration of polymer chains and leading to the presence of an exothermic peak of cold crystallization around 110.3 °C for the PLA. The exothermic peak of cold crystallization of the PLA/TPU blends decreases with the increase of the TPU fraction, and no exothermic peak of cold crystallization is present for the TPU, as expected.

**Figure 3:**
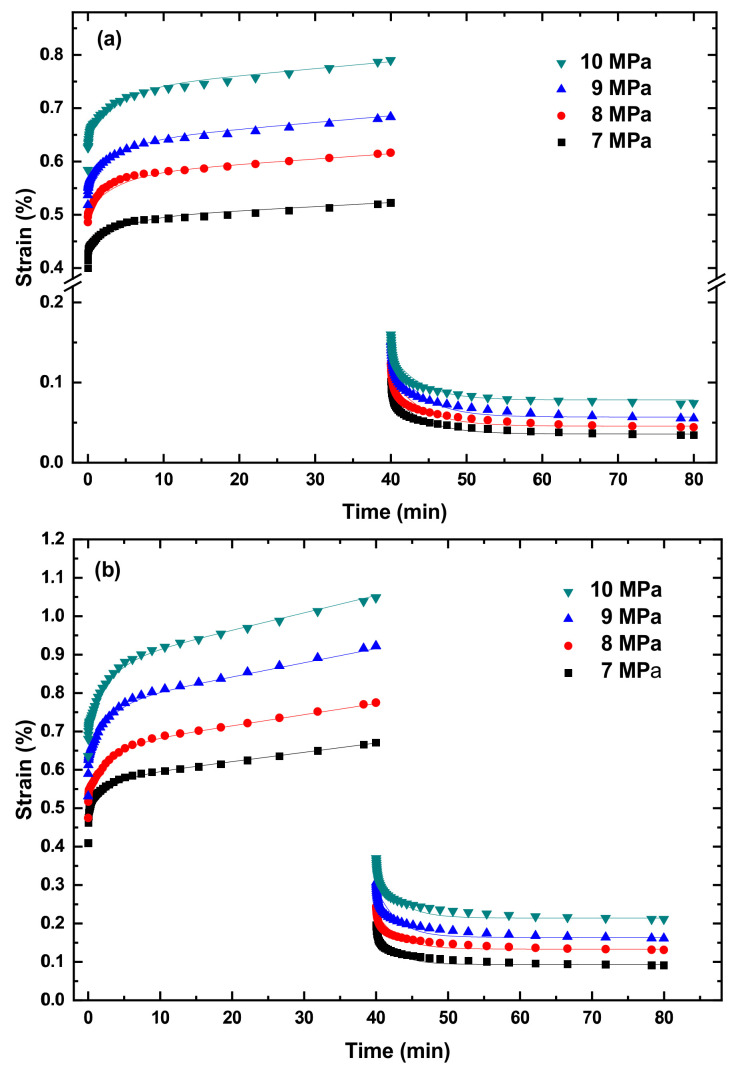

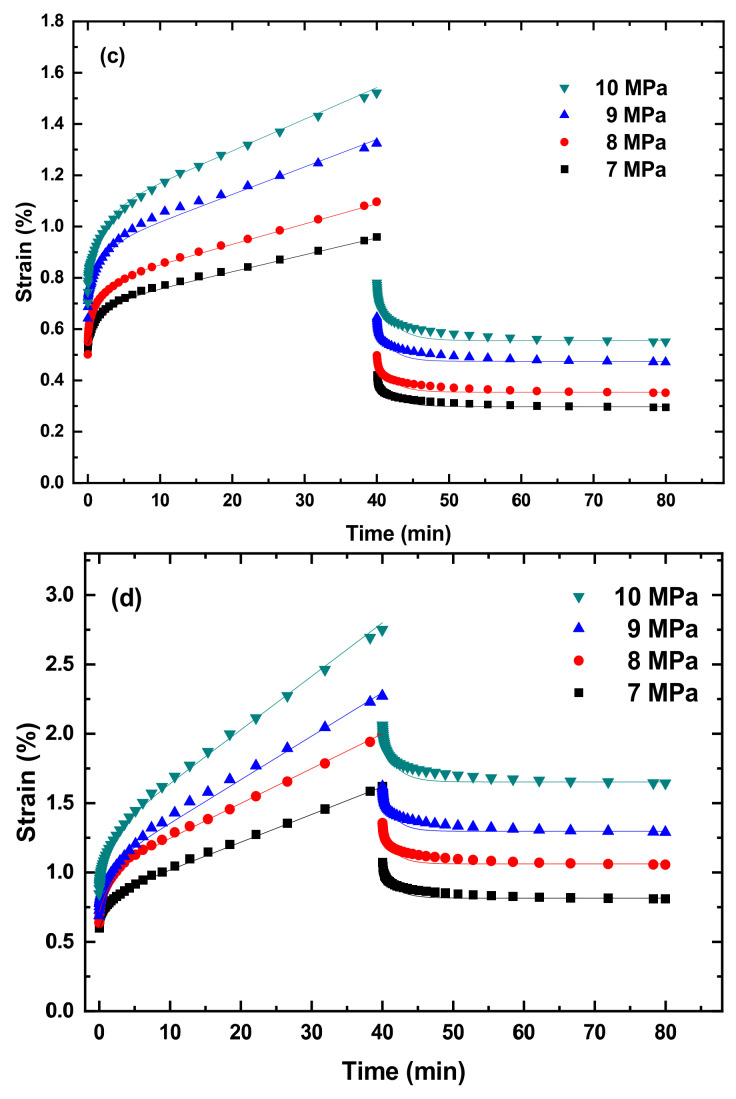
Creep curves of the PLA/TPU blend with 70:30 for the mass ratio of PLA to TPU under different tensile stresses at temperatures of (**a**) 10 °C, (**b**) 20 °C, (**c**) 30 °C, and (**d**) 40 °C.

Figure 3 and Figures S1–S5 in Supplementary Information present the creep curves and recovery curves of the PLA, TPU, and PLA/TPU blends under different tensile stresses at four temperatures of 10, 20, 30, and 40 °C. It is evident that the creep curves consist of two states—a transient state and a steady state (secondary creep). There is a recovery process after the complete removal of the stress/load for all the PLA, TPU, and PLA/TPU blends. It should be noted that the creep deformation of all the PLA, TPU, and PLA/TPU blends was confined to the secondary creep to avoid the presence of tertiary creep and the failure/breakage of the specimens.

## 4. Discussion

**Figure 4:**
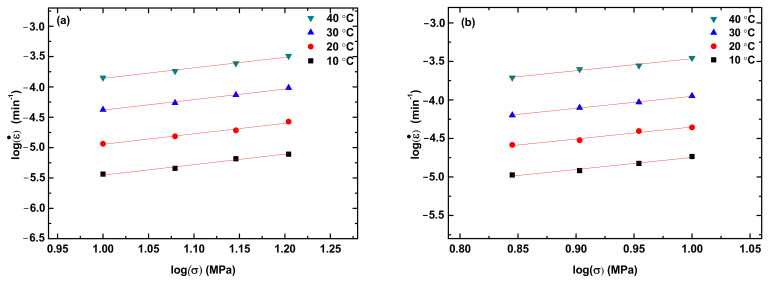

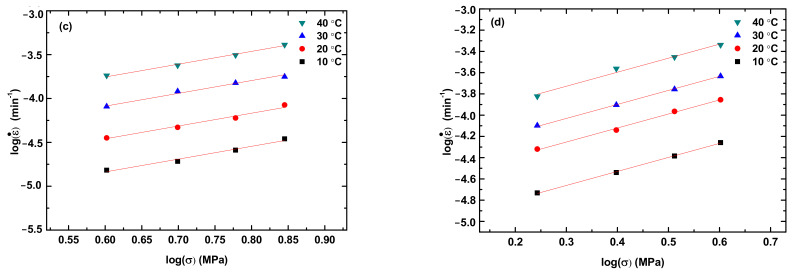
Stress dependence of the creep rate at the steady-state creep for different creep temperatures: (**a**) PLA, (**b**) PLA/TPU blend with 70:30 in the ratio of PLA to TPU, (**c**) PLA/TPU blend with 50:50 in the ratio of PLA to TPU and (**d**) PLA/TPU blend with 30:70 in the ratio of PLA to TPU.

From Figure 3 and Figures S1–S5, we calculate the creep rate at the steady state. Figure 4 shows the variation of the creep rate with the applied stress for the steady state creep of the PLA, PLA/TPU blends, and TPU at different creep temperatures. The creep rate at the steady state increases with the increases in temperature and applied stress, as expected.

In general, the correlation between stress and creep rate at the steady-state creep can be expressed as a power-law law as [28]

(1) $$\dot{\varepsilon }={A\sigma }^{n}$$

where $\dot{\varepsilon }$ is the creep rate, A is a pre-exponential constant, σ is the applied stress, and n is the stress exponent. Using Equation (1) to fit the experimental data in Figure 4, we obtain the stress exponent n. For comparison, the fitting curves are included in Figure 4. It is evident that Equation (1) describes well the correlation between the applied stress and the creep rate at the steady-state creep and there is no statistical difference in the stress exponent for the same material. Note that Equation (1) can be used to determine the activation energy for the creep at different temperatures under the same stress/load. For polymer, however, the creep deformation is better described by viscoelasticity as discussed below.

**Figure 5:**
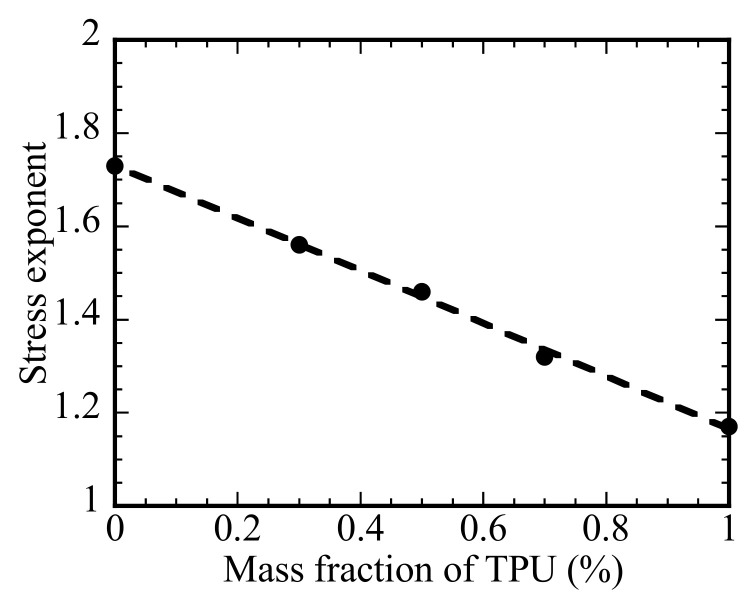
Variation of the stress exponent with the mass fraction of TPU.

Figure 5 displays the variation of the stress exponent with the mass fraction of TPU. The stress exponent decreases linearly from 1.73 for the PLA to 1.17 for the TPU. Such a trend suggests that increasing the fraction of TPU in the PLA/TPU blend reduces the resistance to the motion of the polymer chains. According to the result in Figure 5, the dependence of the stress exponent on the mass fraction of TPU in the PLA/TPU blend can be expressed as

n = 1.73 − 1.17m_TPU_/(m_PLA_ + m_TPU_) (2)

where m_PLA_ and m_TPU_ are the masses of the PLA and TPU, respectively. Such a result is consistent with that PLA and TPU are thermodynamically immiscible.

**Figure 6:**
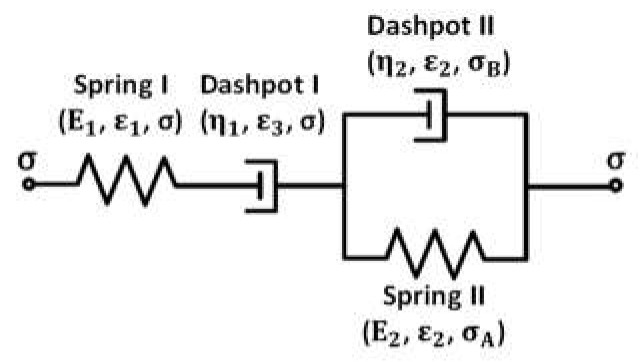
Kelvin representation of non-linear Burgers model.

Figure 6 shows the schematic of Kelvin representation of the non-linear Burgers model. Here, E_1_ and E_2_ are elastic constants of the corresponding spring elements, η_1_ is the viscosity for nonlinear dashpot I, η_2_ is the viscosity of the linear dashpot II, σ, σ_A_, and σ_B_ are the stresses acting on the corresponding elements, and ε, ε_1_ and ε_2_ are the strain of the corresponding elements. The springs I and II and dashpot II are linear elements.

To analyze the creep deformation of the PLA, TPU, and PLA/TPU blends with the power-law relation between the stress and creep rate at the steady-state creep, we introduce the Kelvin representation of the non-linear Burgers model, as shown in Figure 6, in which the stress dependence of the creep rate of the dashpot I follows a power-law relation similar to Equation (1) as

(3) $$\sigma^{n}=\eta_{1}{\dot{\varepsilon }}_{3}$$

The strain/strain rate that other elements experience is proportional to the corresponding applied stress. Under the action of constant stress (creep deformation), we can follow the same approach as the Kelvin representation of the linear Burgers model to obtain the time dependence of the resultant strain, ε, of the non-linear Burgers model as

(4) $$\varepsilon =\frac{\sigma }{E_{1}}+\frac{\sigma^{n}t}{\eta_{1}}+\frac{\sigma }{E_{2}}\left[1-\exp\left(-\frac{E_{2}t}{\eta_{2}}\right)\right]=\frac{\sigma }{E_{1}}+\frac{\sigma^{n}t}{\eta_{1}}+\frac{\sigma }{E_{2}}\left[1-\exp\left(-\beta_{c}t\right)\right]$$

with t being the creep time, and β_c_ = E_2_/η_2_. Here, E_1_ and η_1_ are the elastic constant of spring 1 and the viscosity of non-linear dashpot 1, respectively, E_2_ and η_2_ are the elastic constant of spring 2 and the viscosity of linear dashpot 2, and $\beta_{c}^{-1}$ is the characteristic time of the non-linear Burgers model for creep deformation. The first term represents instantaneous elastic deformation, the second term represents steady-state creep, and the third term corresponds to transient creep deformation.

Using the exponents presented in Figure 5 and Equation (4), we curve-fit the creep curves in Figure 3 and Figures S1–S5 and determine the parameters of E_1_, E_2_, η_1_ and η_2_. For comparison, the fitting curves are included in Figure 3 and Figures S1–S5. It is evident that Equation (4) describes well the creep deformation of the PLA, TPU, and PLA/TPU blends up to the steady-state creep.

Table 1 summarizes the temperature dependence of the elastic constants of the PLA, TPU, and PLA/TPU blends. Both E_1_ and E_2_ of the material decrease with the increase of the creep temperature, as expected, which is due to the increase in space allowing less constraint to the stretch of polymer chains. According to Table 1, increasing the mass fraction of TPU causes decreases in both E_1_ and E_2_, which is due to that the PLA has a higher modulus of 2.382 ± 0.114 GPa than 0.025 ± 0.003 GPa of the TPU.

**Figure 7:**
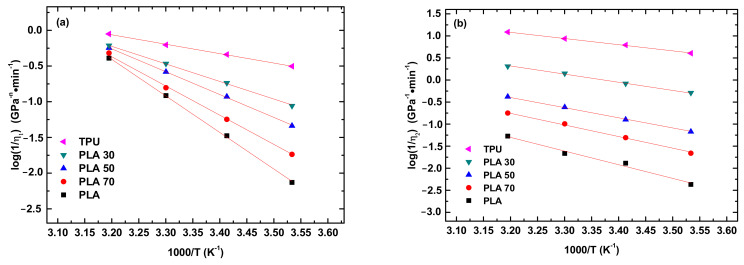
Temperature dependence of η_1_ (**a**) and η_2_ (**b**).

According to the theory of the thermal activation process, the temperature dependence of η_1_ and η_2_ follows the Arrhenius relation as

(5) $$\eta_{1}^{-1}=\eta_{10}^{-1}\exp\left(-\frac{Q_{s}}{\mathrm{RT}}\right)\;\mathrm{and}\;\eta_{2}^{-1}=\eta_{20}^{-1}\exp\left(-\frac{Q_{k}}{\mathrm{RT}}\right)$$

where $\eta_{10}$ and $\eta_{20}$ are two constants, Q_s_ and Q_k_ are the activation energies of the rate processes for the steady-state creep and transient creep, respectively, R is the gas constant, and T is the absolute temperature. Figure 7 shows the temperature dependence of η_1_ and η_2_. It is evident that both the $\eta_{1}^{-1}$ and $\eta_{2}^{-1}$ are exponentially decreasing functions of T^−1^. Using Equation(5) to fit the data in Figure 7a,b, we obtain the activation energies for the creep deformation of the PLA, TPU, and PLA/TPU blends.

**Figure 8:**
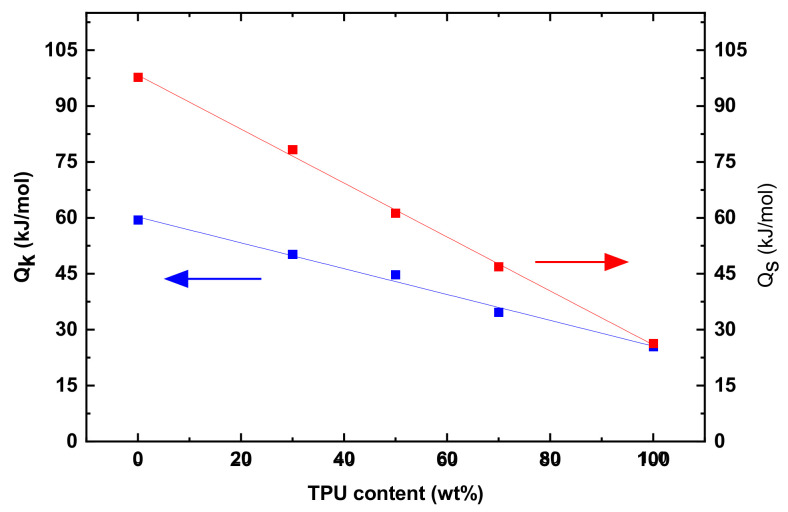
Variations of Q_s_ and Q_k_ with the mass fraction of TPU.

Figure 8 displays variations of Q_s_ and Q_k_ with the mass fraction of TPU. Both the Q_s_ and Q_k_ decrease linearly with the increase of the mass fraction of TPU, suggesting that the activation energies of the PLA/TPU blends can be expressed as

(6) $$\left(Q_{s}^{b},\;Q_{k}^{b}\right)=\left(Q_{s}^{PLA},\;Q_{k}^{PLA}\right)-\left(Q_{s}^{TPU},\;Q_{k}^{TPU}\right)\;\mathrm{mTPU}/\left(\mathrm{mPLA}\;+\;\mathrm{mTPU}\right)$$

in which the superscript b represents the PLA/TPU blend, and the superscript and subscript of PLA and TPU represent the corresponding PLA and TPU. Such a result is consistent again with that PLA and TPU are thermodynamically immiscible. Note that Q_s_ is larger than Q_k_ for the same PLA/TPU blend. Such behavior is associated with the different states of the polymer chains. Before the onset of the steady-state creep, the polymer chains are in a relatively relaxed state with less resistance to migration. At the steady state creep, the polymer chains are under stretch with a large resistance to the migration. The polymer chains need to overcome a larger energy barrier to reach a new state at the steady state creep than at the transient state.

According to Figure 3 and Figures S1–S5, the PLA/TPU blends experienced an instantaneous elastic recovery, then a time-related recovery, and finally a permanent plastic deformation after the end of the creep deformation. Following the analysis of the creep deformation of the PLA/TPU blends, we use the Kelvin representation of the non-linear Burgers model to analyze the recovery behavior of the PLA/TPU blends. The time dependence of the recovery strain is expressed as

(7) $$\varepsilon_{r}\left(t\right)=\left\{\left[\varepsilon \left(t_{c}\right)\right]-\frac{\sigma }{E_{1}}-\varepsilon_{p}\right\}\exp\left[-\frac{E_{2}\left(t-t_{c}\right)}{\eta_{2}}\right]+\varepsilon_{p}\;\mathrm{with}\;\varepsilon_{p}={B\sigma }^{m}\;\exp\left(-\frac{Q_{p}}{\mathrm{RT}}\right)$$

where $t_{c}$ is the creep time of the creep test, $\varepsilon \left(t_{c}\right)$ is the final strain of the creep deformation, and $\varepsilon_{p}$ is the plastic strain. For the plastic strain, B is a pre-exponential constant, m is the stress exponent, and $Q_{p}$ is the activation energy of the plastic deformation. Using the first equation in Equation (7) to curve-fit the recovery curves, we obtain the plastic strain. Figures S6 and S7 in Supplementary Information display the stress dependence of the plastic strain for the recovery of the PLA/TPU blends at different temperatures and the temperature dependence of the plastic strain for the recovery of the PLA/TPU blends under different stresses, respectively. Using the second equation in Equation (7) to curve-fit the results in Figures S6 and S7, we determine the stress exponents and activation energies, as listed in Table 2. Both the stress exponent (m) and the activation energy (Qp) are the same as the corresponding ones (n and Q_s_) for the steady-state creep of the same PLA/TPU blends. Such results reveal the same rate mechanisms controlling the migration of polymer chains at the steady-state creep and the recovery after the creep deformation.

## 5. Conclusions

In summary, we have studied the thermal behavior, tensile creep deformation and recovery after complete unloading of the PLA, TPU, and PLA/TPU blends under different stresses in the temperature range of 10 to 40 °C. The thermal analysis has revealed that the PLA/TPU blends maintained the thermal characteristics of individual PLA and TPU, consistence with that PLA and TPU are thermodynamically immiscible. To avoid the presence of tertiary creep and the failure/breakage of the specimens, we have focused on the creep deformation of the PLA, TPU, and PLA/TPU blends on the transient and steady-state creep. The stress dependence of the creep rate of the PLA, TPU, and PLA/TPU blends for the steady-state creep under the conditions used in this work follows a power-law relation. The stress exponent of the power-law relation is a linearly decreasing function of the mass fraction of TPU in consistence with that PLA and TPU are thermodynamically immiscible.

On the base of the power-law relation between the tensile stress and creep rate at a steady state, we have proposed a Kelvin representation of the non-linear Burgers model for the analysis of the creep deformation and recovery of the PLA, TPU, and PLA/TPU blends. Such a Kelvin representation of the non-linear Burgers model can describe well the creep deformation and recovery of the PLA, TPU, and PLA/TPU blends. The results obtained from the curve-fitting of the creep and recovery curves reveal that the stress exponent and activation energies are linearly decreasing functions of the mass fraction of TPU, which are consistent with that PLA and TPU are thermodynamically immiscible. The activation energy of the transient creep is less than that of the steady state creep for the same PLA/TPU blend, revealing the increase in the resistance to the migration of polymer chains at the steady-state creep.

## Figures and Tables

**Table 1:** Temperature dependence of E_1_ and E_2_ of the PLA, TPU, and PLA/TPU blends.

Material	Modulus	Temperature (°C)			
		10	20	30	40
PLA	E_1_ (GPa)	2.65 ± 0.07	2.53 ± 0.06	2.41 ± 0.06	2.31 ± 0.07
	E_2_ (GPa)	38.6 ± 2.0	23.4 ± 0.7	18.6 ± 0.9	11.2 ± 0.5
PLA70/TPU30	E_1_ (GPa)	1.59 ± 0.06	1.43 ± 0.05	1.27 ± 0.06	1.15 ± 0.08
	E_2_ (GPa)	11.5 ± 0.7	7.09 ± 0.42	4.61 ± 0.35	2.99 ± 0.24
PLA50/TPU50	E_1_ (GPa)	1.05 ± 0.053	0.929 ± 0.055	0.772 ± 0.023	0.707 ± 0.026
	E_2_ (GPa)	4.97 ± 0.2	3.36 ± 0.28	1.87 ± 0.13	1.28 ± 0.09
PLA30/TPU70	E_1_ (GPa)	0.425 ± 0.021	0.366 ± 0.026	0.345 ± 0.017	0.309 ± 0.02
	E_2_ (GPa)	0.779 ± 0.04	0.516 ± 0.028	0.326 ± 0.021	0.232 ± 0.017
TPU	E_1_ (MPa)	35.6 ± 0.9	30.8 ± 1.2	26.8 ± 0.7	23.9 ± 0.7
	E_2_ (MPa)	103 ± 6	71.0 ± 4.6	51.4 ± 4.1	37.6 ± 2.4

**Table 2:** Numerical values of m and Q_p_ for the recovery of the PLA/TPU blends after the creep.

Material	Temperature (10–40 °C)	
	m	Q_p_ (kJ/mol)
PLA	1.73	96.5 ± 3.1
PLA70/TPU30	1.56	76.8 ± 3.3
PLA50/TPU50	1.46	62.3 ± 2.5
PLA30/TPU70	1.32	45.4 ± 2.7
TPU	1.17	27.4 ± 1.5

## Data Availability

Not applicable.

## Supplementary Materials

The following supporting information can be downloaded at: https://www.mdpi.com/article/10.3390/polym14235276/s1, Figures S1–S5: Creep curves for PLA, PLA70/TPU30, PLA50/TPU50, PLA30/TPU70, and TPU, respectively. Figure S6: Stress dependence of the plastic strain at different creep temperatures for the recovery behavior. Figure S7: Temperature dependence of the plastic strain for the recovery behavior.
