# Supplementary material for: Thermo-Mechanical and Creep Behaviour of Polylactic Acid/Thermoplastic Polyurethane Blends
Source: Polymers (Basel). 2022 Dec 2;14(23):5276. doi: 10.3390/polym14235276 (PMC9738534; doi:10.3390/polym14235276)
Supplement: Supplementary file 1 [file polymers-14-05276-s001.zip › polymers-1965933-supplementary.pdf]

polymers

MDPI

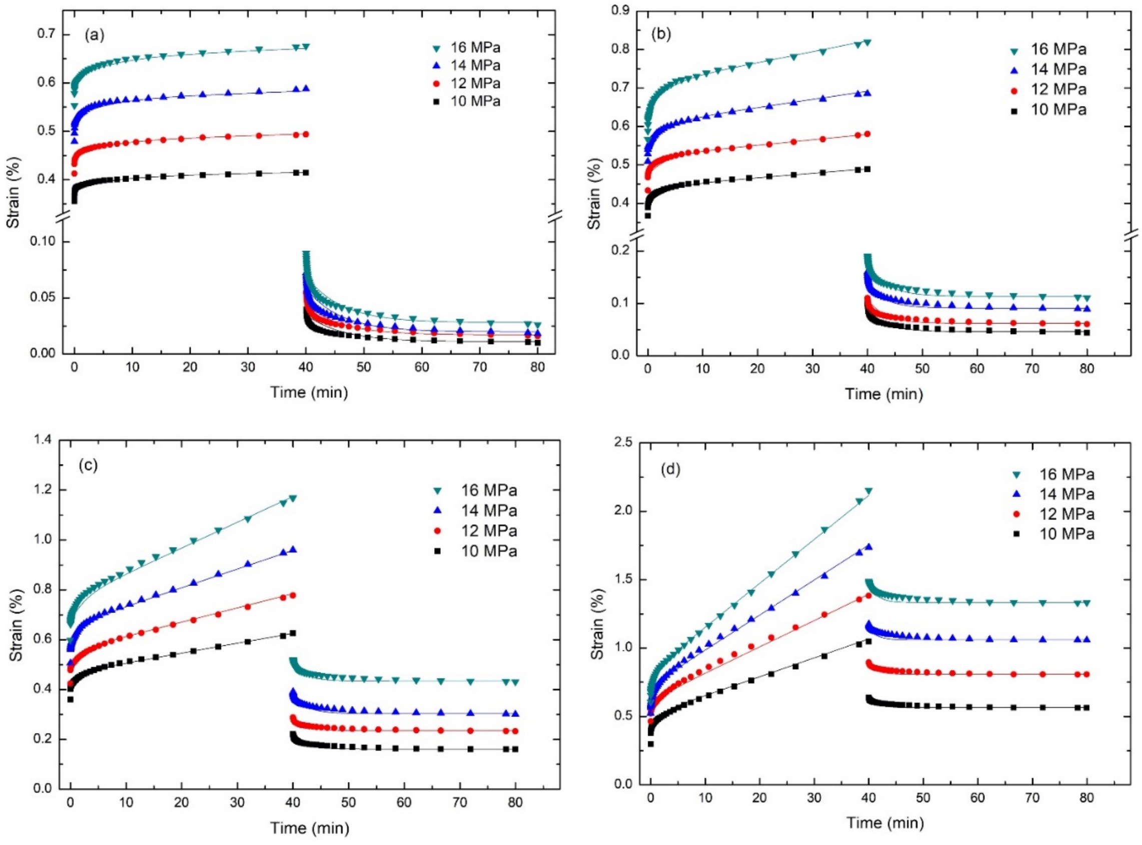

Figure S1. Creep curves for PLA under different stresses at different temperatures: (**a**) 10 ℃ (**b**) 20 ℃ (**c**) 30 ℃ (**d**) 40 ℃.

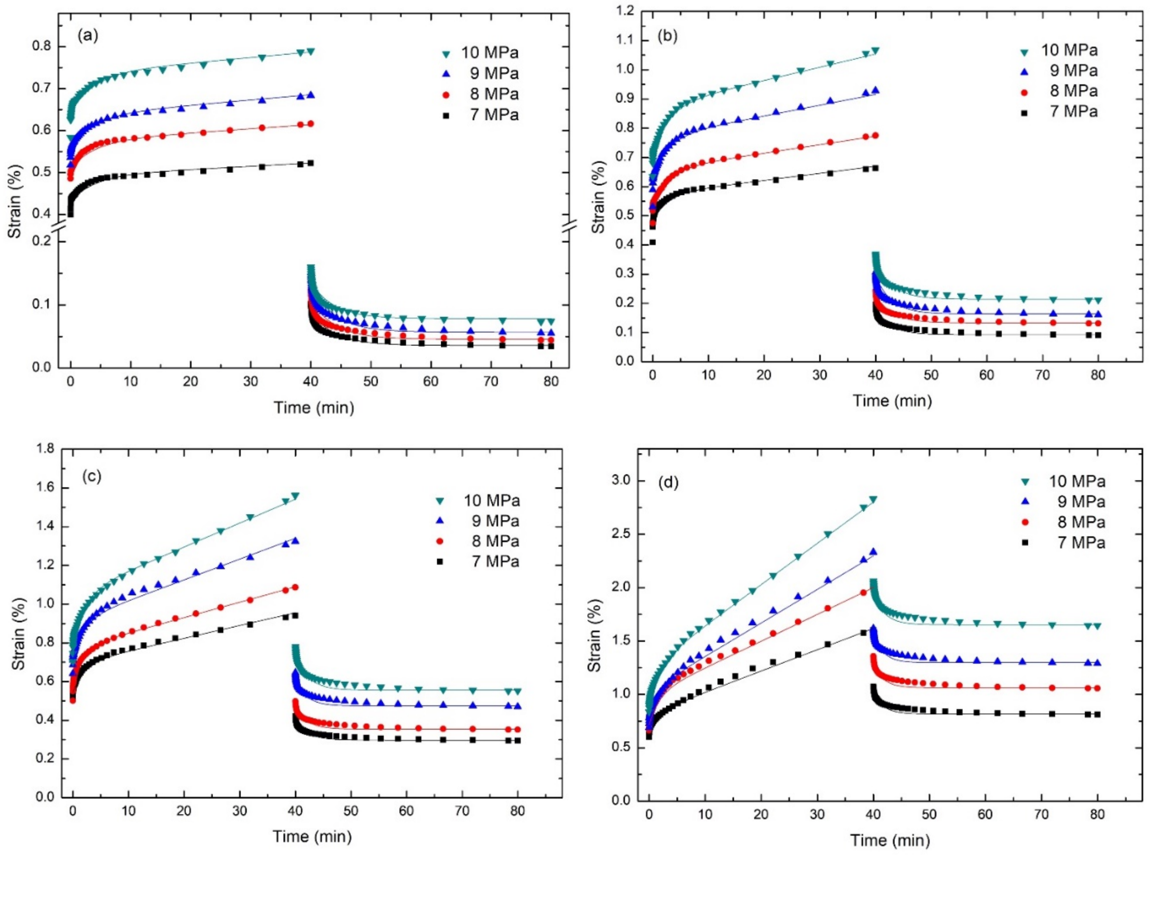

Figure S2. Creep curves for PLA70/TPU30 under different stresses at different temperatures: (**a**) 10 ℃ (**b**) 20 ℃ (**c**) 30 ℃ (**d**) 40 ℃.

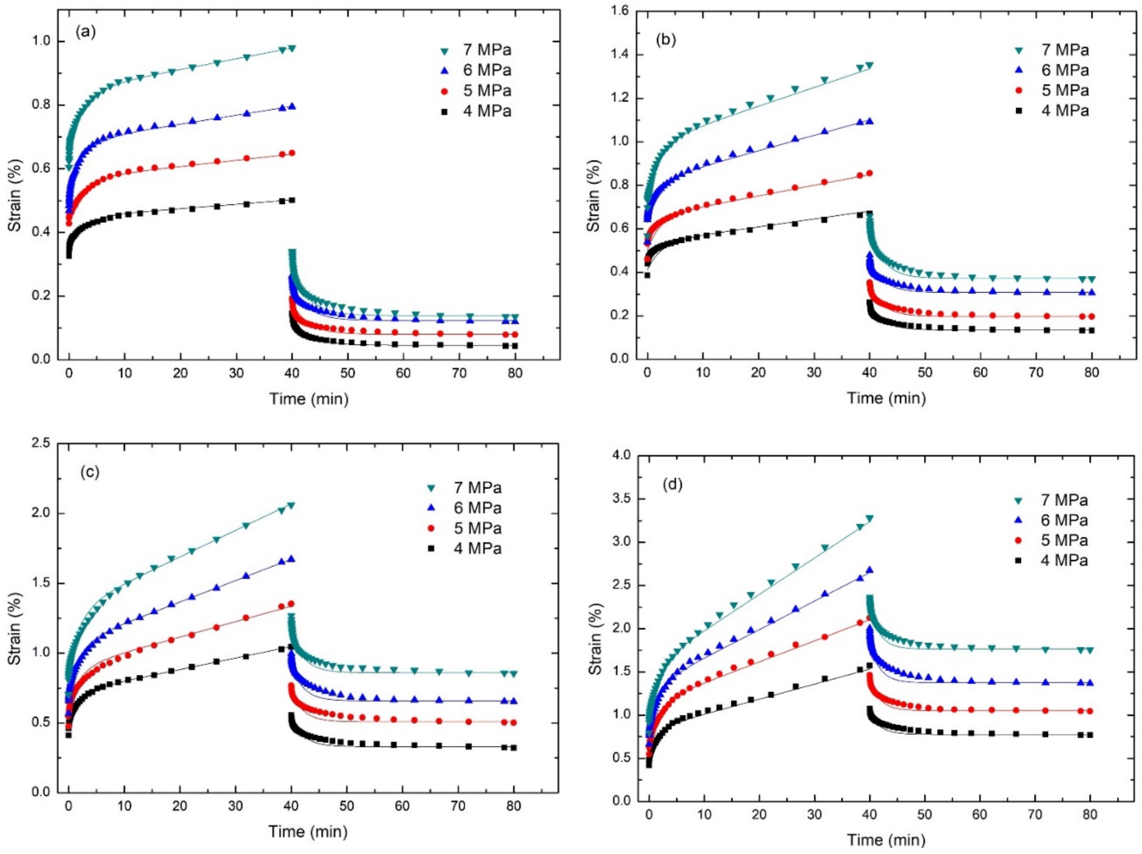

Figure S3. Creep curves for PLA50/TPU50 under different stresses at different temperatures: (**a**) 10 ℃ (**b**) 20 ℃ (**c**) 30 ℃ (**d**) 40 ℃.

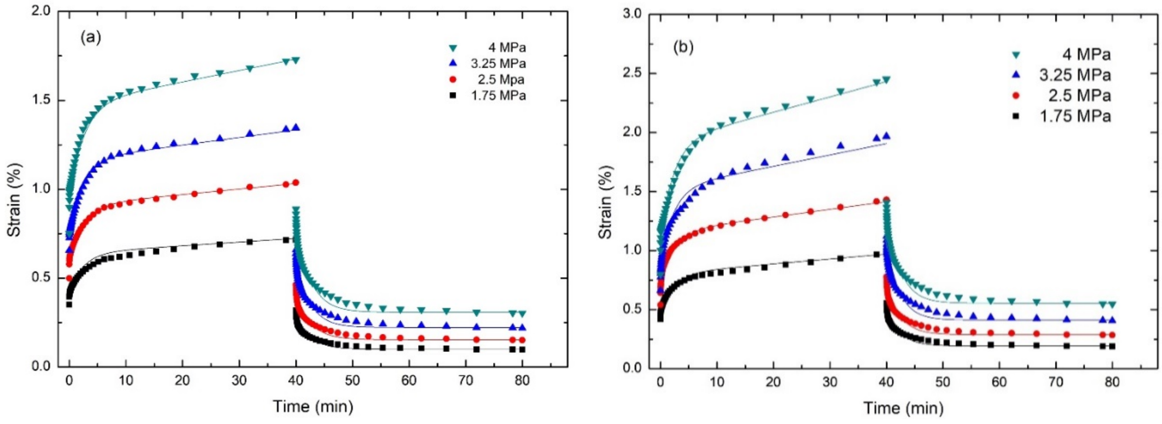

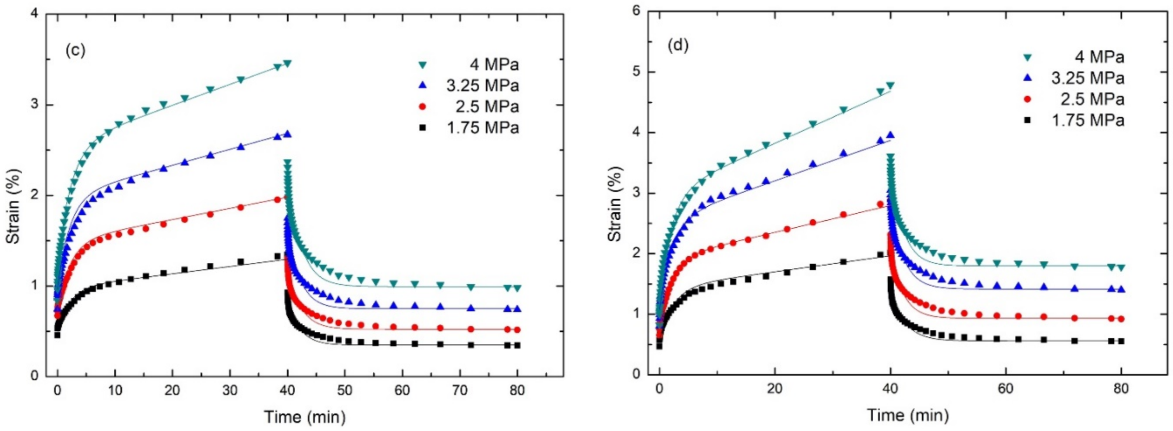

Figure S4. Creep curves for PLA30/TPU70 under different stresses at different temperatures: (**a**) 10 ℃ (**b**) 20 ℃ (**c**) 30 ℃ (**d**) 40 ℃.

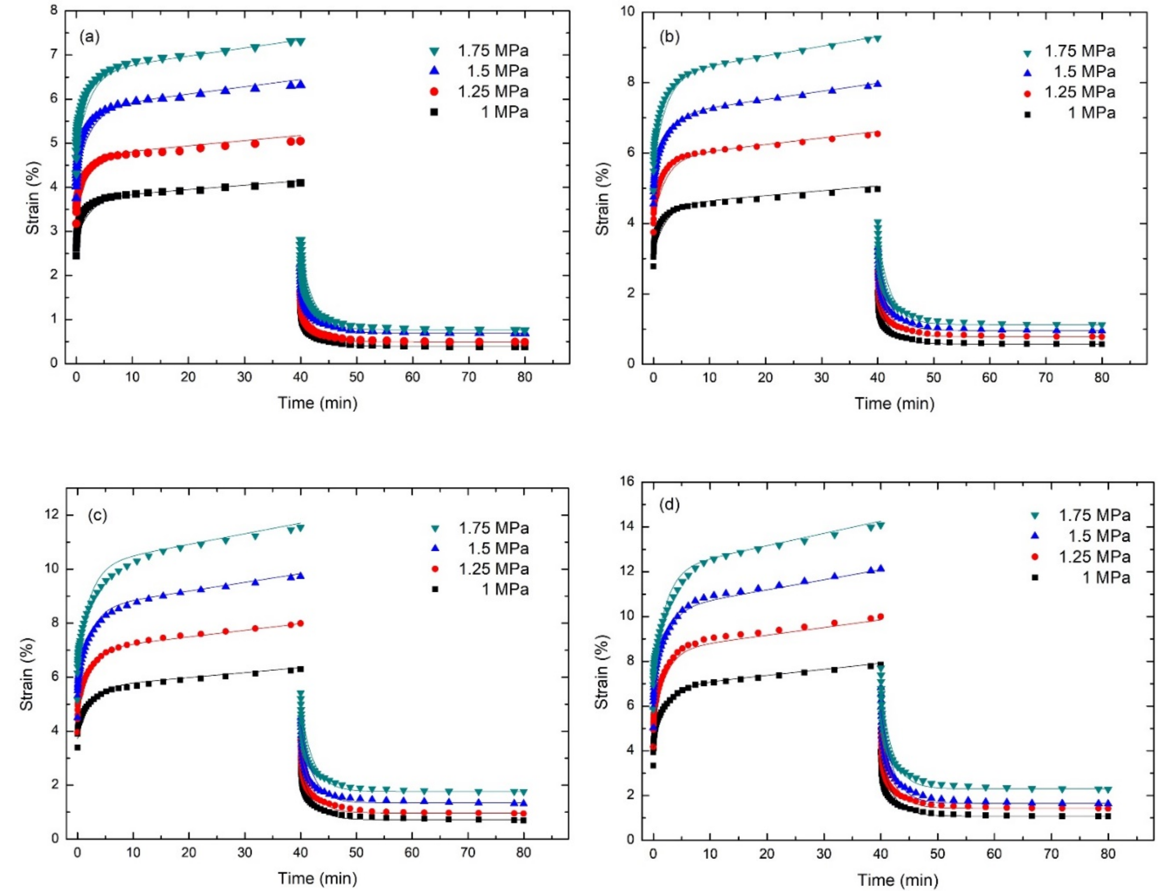

Figure S5. Creep curves for TPU under different stresses at different temperatures: (**a**) 10 ℃ (**b**) 20 ℃ (**c**) 30 ℃ (**d**) 40 ℃.

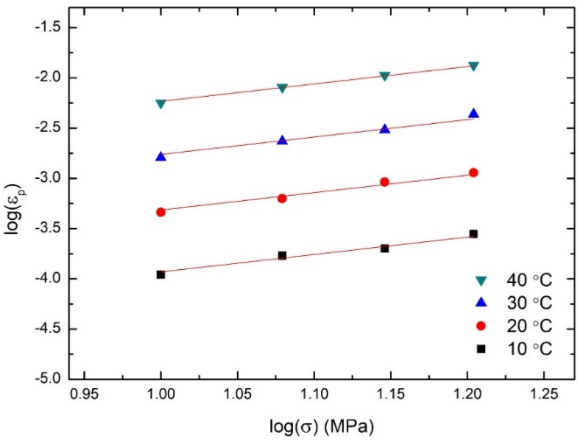
log(εp)
log(σ) (MPa)
40 °C
30 °C
20 °C
10 °C

(a)

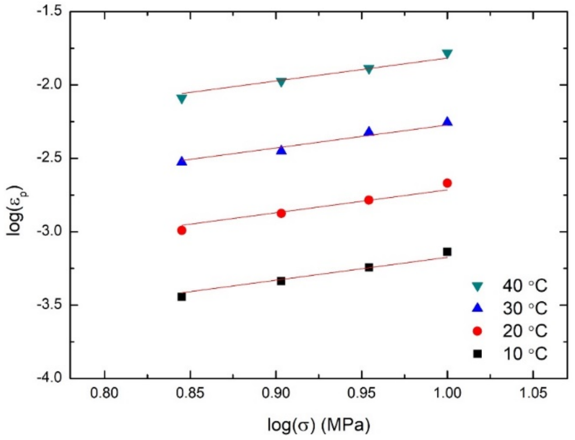
log(εp)
log(σ) (MPa)
40 °C
30 °C
20 °C
10 °C

(b)

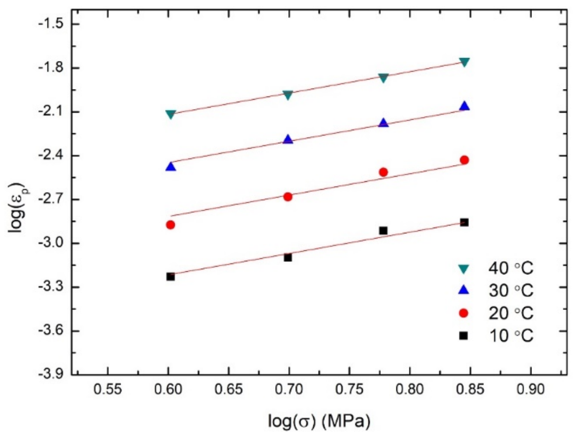
log(εp)
log(σ) (MPa)
40 °C
30 °C
20 °C
10 °C

(c)

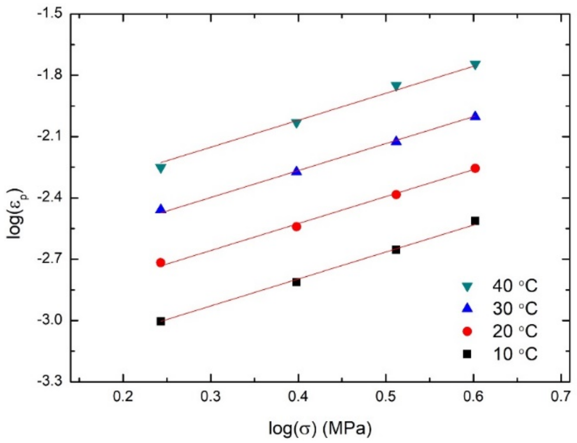
log(εp)
log(σ) (MPa)
40 °C
30 °C
20 °C
10 °C

(d)

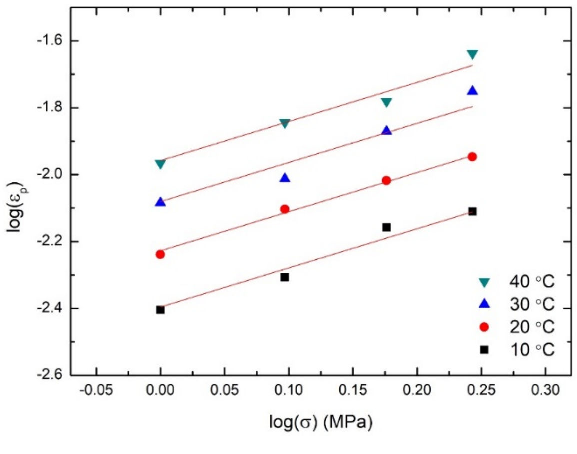
log(εp)
log(σ) (MPa)
40 °C
30 °C
20 °C
10 °C

(e)

Figure S6. Stress dependence of the plastic strain at different creep temperatures for the recovery of (**a**) PLA, (**b**) PLA/TPU blend with 70:30 for the ratio of PLA to TPU, (**c**) PLA/TPU blend with 50:50 for the ratio of PLA to TPU, (**d**) PLA/TPU blend with 30:70 for the ratio of PLA to TPU, and (e) TPU.

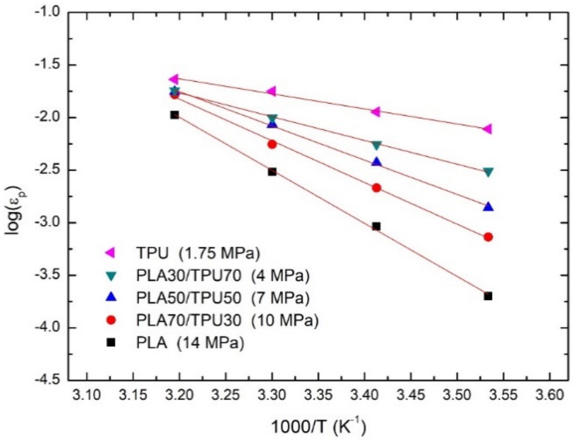

Figure S7. Temperature dependence of the plastic strain for the recovery of the PLA/TPU blends under different stresses.
